# Characterization of Samples Identified as Hepatitis C Virus Genotype 1 without Subtype by Abbott RealTi*m*e HCV Genotype II Assay Using the New Abbott HCV Genotype *Plus* RUO Test

**DOI:** 10.1128/JCM.02264-15

**Published:** 2016-01-28

**Authors:** Camelia Mokhtari, Anne Ebel, Birgit Reinhardt, Sandra Merlin, Stéphanie Proust, Anne-Marie Roque-Afonso

**Affiliations:** a Virologie AP-HP Hôpital Paul Brousse, Villejuif, France; b Biomnis, Ivry sur Seine, France; c Abbott GmbH & Co. KG, Wiesbaden, Germany

## Abstract

Hepatitis C virus (HCV) genotyping continues to be relevant for therapeutic strategies. Some samples are reported as genotype 1 (gt 1) without subtype by the Abbott RealTi*m*e HCV Genotype II (GT II) test. To characterize such samples further, the Abbott HCV Genotype *Plus* RUO (Plus) assay, which targets the core region for gt 1a, gt 1b, and gt 6 detection, was evaluated as a reflex test in reference to NS5B or 5′-untranslated region (UTR)/core region sequencing. Of 3,626 routine samples, results of gt 1 without subtype were received for 171 samples (4.7%), accounting for 11.5% of gt 1 specimens. The Plus assay and sequencing were applied to 98 of those samples. NS5B or 5′-UTR/core region sequencing was successful for 91/98 specimens (92.9%). Plus assay and sequencing results were concordant for 87.9% of specimens (80/91 samples). Sequencing confirmed Plus assay results for 82.6%, 85.7%, 100%, and 89.3% of gt 1a, gt 1b, gt 6, and non-gt 1a/1b/6 results, respectively. Notably, 12 gt 6 samples that had been identified previously as gt 1 without subtype were assigned correctly here; for 25/28 samples reported as “not detected” by the Plus assay, sequencing identified the samples as gt 1 with subtypes other than 1a/1b. The genetic variability of HCV continues to present challenges for the current genotyping platforms regardless of the applied methodology. Samples identified by the GT II assay as gt 1 without subtype can be further resolved and reliably characterized by the new Plus assay.

## INTRODUCTION

It is estimated that more than 185 million people have been infected with hepatitis C virus (HCV) (1). Persistent infection develops in 60 to 85% of cases and is a leading cause of chronic hepatitis, cirrhosis, and liver cancer worldwide (2). Through phylogenetic analysis, 7 genotypes (gts) and 67 confirmed subtypes have been described (3). Genotype 1 is the most prevalent genotype worldwide, corresponding to almost 50% of HCV cases (4). The rapid development of safe effective antiviral drugs that directly target HCV offers hope for HCV eradication (5). However, it has been observed that the efficacy and barrier to resistance of several new direct-acting antiviral agents (DAAs) could depend on the HCV genotype and the HCV gt 1 subtype (6). Therefore, accurate genotyping methods are required for appropriate treatment implementation.

Several molecular methods can be used to identify HCV genotypes and subtypes in clinical practice. Nucleotide sequencing followed by phylogenetic analysis of variable regions of the HCV genome, such as the core/E1 or NS5B region, has been recommended in consensus proposals (7), but amplification of these highly variable regions may be difficult and relies on laboratory-developed tests with time-consuming techniques that are not readily adapted for routine laboratory practice. The 5′ untranslated region (UTR), a highly conserved part of the HCV genome, is used by diagnostic HCV RNA assays and also is a common target for HCV genotyping by most commercial assays. These assays have been shown to differentiate well between most HCV genotypes. However, the 5′ UTR is less appropriate for discriminating between gt 1 subtypes 1a and 1b (8, 9). Therefore, the RealTi*m*e HCV Genotype II (GT II) assay (Abbott Molecular Inc., Des Plaines, IL, USA), a real-time reverse transcription (RT)-PCR-based assay, uses genotype- and subtype-specific primers and probes targeting the 5′ UTR for genotyping and NS5B for subtype 1a and 1b identification.

As reported, however, no subtype 1a or 1b could be identified for 3.7 to 15.9% of gt 1 results (10–15). Those studies reported gt 1 results with either delayed or missing gt 1a or 1b amplification curves. The results without 1a/1b amplification curves might indicate a gt 1 subtype other than 1a or 1b or gt 6 subtypes with 5′-UTR sequences identical to that of subtype 1b (14). To characterize these samples further, the manufacturer has developed a new assay, the Abbott HCV Genotype *Plus* RUO (Plus) assay, targeting the core region and specifically identifying genotypes 1a, 1b, and 6. The present study analyzes the performance of this new assay as a supplementary test for samples with gt 1 results without subtype identification, in reference to NS5B and 5′-UTR/core region sequencing.

## MATERIALS AND METHODS

### Patient samples.

A total of 3,626 routine HCV genotype analyses were performed between April 2013 and June 2014 by the Biomnis laboratory using the GT II assay, according to the manufacturer's instructions. Among these specimens, 1,306 were identified as gt 1a or gt 1b (36%) and 171 (4.7%) returned a gt 1 result without subtype, representing 11.6% of all gt 1 results. Of these, 40 samples (23.4%) presented with delayed gt 1a or gt 1b amplification curves, which appeared too late to be captured by the software algorithm, and 131 samples (76.6%) showed no subtype amplification curves. Based on available archived residual specimens, 98/171 samples with gt 1 results without subtype were subjected to the Plus assay and later to NS5B and/or 5′-UTR/core region sequencing at the virology laboratory of the Paul Brousse Hospital.

### Abbott HCV Genotype *Plus* RUO assay.

The Plus assay (Abbott Molecular Inc., Des Plaines, IL, USA) uses a multiplex RT-PCR to generate HCV RNA amplicons from clinical specimens. An unrelated RNA sequence serves as an internal control (IC) and is simultaneously extracted and amplified to verify the correct processing of each individual sample. The assay selectively detects HCV subtypes 1a and 1b and genotype 6 in a single reaction, using specific fluorescently labeled oligonucleotide probes targeting the HCV core region. The assay is fully automated; the Abbott *m*2000sp platform performs HCV RNA extraction, mastermix preparation, and PCR plate setup, while the Abbott *m*2000rt instrument is used for amplification and detection. The Abbott *m*2000rt instrument automatically reports, on the Abbott *m*2000rt workstation, the genotype call results as gt 1a, gt 1b, gt 6, or “not detected,” which could correspond to a gt 1 subtype other than 1a or 1b, as only samples with gt 1 results from the GT II test were subjected to the Plus test in the present study.

### Sequencing analysis.

Specimens were sequenced primarily in the NS5B region. If amplification of this region failed, then the 5′ UTR/core region was sequenced instead. RT-PCR amplifications of the NS5B region and 5′ UTR/core region were performed with a one-step protocol using the Qiagen OneStep RT-PCR kit (Qiagen, Courtaboeuf, France), with the primers listed in Table 1. Bidirectional sequencing of the amplicons was performed using a BigDye Terminator version 3 ready reaction cycle sequencing kit (Applied Biosystems, Foster City, CA) on an Applied Biosystems 3130 automatic sequencer, according to the manufacturer's protocol.

**TABLE 1:** Primers used for sequence analysis

Primer target and sense	Sequence
5′ UTR/core region	
Forward	GCAGAAAGCGTCTAGCCATGGCGT
Reverse	CGCGCGCACACCCAATCTRGGG
NS5B	
Forward	TATGAYACCCGCTGYTTTGACTC
Reverse	GCXGARTAYCTVGTCATAGCCTC

Phylogenetic analysis was performed after sequence alignment with reference sequences of genotypes 1 to 7, using the neighbor-joining method in MEGA5 software. The reliability of the phylogenetic clustering was demonstrated using bootstrap analysis with 1,000 replicates. BLAST searches were performed in HCV sequence databases (http://hcv.lanl.gov and http://comet.retrovirology.lu/hcv).

## RESULTS

In our study, 98 routine samples that had been identified by the GT II assay as gt 1 without subtype assignment were retested using the Plus assay and NS5B and/or 5′-UTR/core region sequencing. NS5B sequencing was successful for 86/98 samples (87.8%), and 5′-UTR/core region sequences could be obtained for 5 of the 12 remaining samples. Thus, the combined overall sequencing efficiency was 92.9% in this study. The concordance analysis between the Plus assay and the sequencing method was based on 91 sequencing results.

As shown in Table 2, among 15 samples with delayed gt 1a amplification curves, all were assigned to gt 1a by the Plus assay, with 92.3% (12/13 samples) of results being confirmed by sequencing. Sequencing failures (both NS5B and 5′ UTR/core regions) were observed for two samples. Among 14 samples with delayed gt 1b amplification curves, 12 were assigned to gt 1b by the Plus test, and results were confirmed by sequencing in 91.7% of cases (11/12 samples). The results for the two remaining samples were reported as not detected by the Plus assay, indicating that genotypes 1a, 1b, and 6 were not present. Sequencing analysis confirmed a gt 1d result for one of the two samples. Therefore, the overall concordance between Plus test and sequencing results was 85.7% (12/14 samples) for samples with delayed 1b amplification curves. Finally, among 69 samples with no subtype amplification curves, the Plus assay results were confirmed by sequencing in 87.5% of cases (56/64 samples), as follows. Eleven samples were assigned to gt 1a by the Plus test, and results were confirmed by sequencing in 70% of cases (7/10 samples); 1 sample failed to yield a sequencing result. Sixteen samples were assigned to gt 1b, and results were confirmed by sequencing in 81.3% of cases (13/16 samples). Twelve samples were assigned to gt 6, and all results were confirmed by sequencing. Thirty samples yielded results of not detected (corresponding to non-1a/1b/6), and results were confirmed as non-1a/1b gt 1 in 92.3% of cases (24/26 samples); no sequencing results could be obtained for the remaining 4 samples.

**TABLE 2:** Concordance of GT II assay, Plus assay, and NS5B or 5′-UTR/core region sequencing results for 98 routine samples with previous GT II assay results of gt 1 without subtype

RealTi*m*e HCV GT II assay result	HCV Genotype *Plus* RUO assay result	No. with NS5B or 5′-UTR/core region sequencing result of					HCV Genotype *Plus* RUO results confirmed by sequencing (no./no. tested [%])
		gt 1b	gt 1a	gt 6	Non-gt 1a/1b/6	No sequence^*a*^	
gt 1 with delayed subtype 1a amplification curve (*n* = 15)	gt 1a	12^*b*^			1^*c*^	2	12/13 (92.3)
gt 1 with delayed subtype 1b amplification curve (*n* = 14)	gt 1b		11^*b*^	1			12/14 (85.7)
	Not detected^*d*^		1		1^*b*^^,^^*c*^		
gt 1 with no subtype amplification curve (*n* = 69)	gt 1a	7^*b*^			3^*c*^	1	56/64 (87.5)
	gt 1b		13^*b*^		3^*e*^		
	gt 6			12^*b*^			
	Not detected^*d*^		2		24^*b*^^,^^*f*^	4	
All gt 1 samples without subtype results (*n* = 98)	gt 1a	19^*b*^			4	3	80/91 (87.9)
	gt 1b		24^*b*^	1	3	0	
	gt 6			12^*b*^		0	
	Not detected^*d*^		3		25^*b*^	4	

a Viral load results were available for 4/7 samples and ranged from 3.78 to 4.51 log IU/ml.

b Concordant results.

c Genotype 1d by sequencing.

d Viral load results were available for 21/32 samples and ranged from 4.41 to 7.1 log IU/ml.

e Genotype 1l by sequencing.

f Genotypes 1c, 1d, 1e (14/24 samples), 1h, and 1l by sequencing.

In summary, for gt 1 samples without subtype 1a or 1b results from the GT II assay, the overall concordance between Plus assay and sequencing results was 87.9% (Table 2). Notably, these samples included 25 samples that were correctly reported as not detected by the Plus assay and were identified by sequencing as gt 1 samples with subtypes other than 1a or 1b. Sequencing confirmed the Plus test results for 82.6%, 85.7%, 100%, and 89.3% of gt 1a, gt 1b, gt 6, and non-gt 1a/1b/6 results, respectively. In particular, 12/12 gt 6 samples that had been classified previously as gt 1 without subtype assignment by the GT II assay were correctly identified as gt 6 by the Plus assay, and results were confirmed by sequencing.

The majority of discordant results between the Plus assay and sequencing (8/10 samples) were classified by the former as gt 1a or gt 1b (4 cases each) but were identified by the latter as gt 1 with subtypes other than 1a or 1b in 7/8 cases and as gt 6 in 1 case. Three samples that were identified as gt 1b by sequencing were missed by the Plus test.

## DISCUSSION

The GT II assay has a number of advantages, such as a high level of automation, genotype and subtype 1a/1b identification, short turnaround times, and ease of result interpretation, and has been reported to genotype and subtype reliably most routine specimens (91.5 to 98%) (10–12, 14, 16). The assay targets the 5′ UTR to identify the genotype, as well as the NS5B region for subtypes 1a and 1b. Due to the high variability of HCV, however, it has been reported that no subtype 1a or 1b was assigned for 3.7 to 15.9% of gt 1 results (10–15). This corresponds to 11.5% of gt 1 results without subtype in the present study. Such results require additional testing, especially if DAA therapy selection relies on subtype 1a/1b classification due to different rates of sustained virological responses and different genetic barriers to resistance (6). Furthermore, rare subtypes of gt 6 and some gt 1b subtypes have identical 5′ UTRs, which makes them indistinguishable by 5′-UTR analysis (14). Hence, a gt 1 result without subtype may also indicate a misclassified gt 6 sample, as observed by Mallory et al., who reported 2 gt 6 samples that had been assigned previously to gt 1 using 5′-UTR sequencing and to gt 1 without subtype identification using the GT II assay (14).

The new Plus assay specifically detects HCV subtypes 1a and 1b and genotype 6 and can be utilized as a reflex assay for gt 1 results without subtype, thus allowing further resolution of unsubtypable results. In total, among the 98 investigated samples identified as genotype 1 without subtype, the Plus test classified 26 samples as gt 1a (26.5%), 28 as gt 1b (28.6%), and 12 as gt 6 (12.2%), while 32 samples yielded a result of not detected (32.7%), indicating that gt 1a, gt 1b, and gt 6 were not present. The latter was supported by viral load results being available for 21 of those 32 samples and ranging from 4.4 to 7.1 log IU/ml, which is above the limit of detection for the Plus assay (data not shown). In fact, sequencing confirmed gt 1 strains with subtypes other than 1a or 1b, such as 1c, 1d, 1e, 1 h, or 1l, for 25 of the 32 samples with results of not detected. Four samples in this group failed to be amplified for sequencing and therefore could not be further characterized. Three samples were classified by sequencing as gt 1b. The reason for these 3 samples being missed by the Plus assay remains unclear, as viral loads were above 5 log IU/ml, which excludes a lack of sensitivity. Since the target region of the assay was outside the sequenced NS5B region, potential polymorphisms could not be verified. In summary, sequencing confirmed the results of the Plus test for 82.6%, 85.7%, 100%, and 89.3% of gt 1a, gt 1b, gt 6, and not detected results, respectively, thus demonstrating that the new assay successfully resolved 87.9% of the previous gt 1 results without subtype.

Generally, accurate identification of genotypes and subtypes 1a and 1b remains challenging for every assay. A previous version of the INNO-LiPA HCV assay suffered from low ability to identify gt 1a and gt 1b, resulting in only 45 to 80% concordance with NS5B sequencing results for gt 1a and gt 1b assignment (8). The performance was enhanced in the second-generation line probe assay, i.e., the INNO-LiPA v2.0 assay (Versant HCV Genotype 2.0 System, Siemens), by the addition of probes targeting the core region (8). Nevertheless, as recently shown by Guelfo et al. (17), the assay still misclassified 15% of gt 1a samples, resulting in overall concordance with core region sequencing results of 89% for nonselected gt 1a and gt 1b samples. Similarly, Cai et al. observed that 52% of gt 6a samples were misclassified as gt 1b samples by the INNO-LiPA v2.0 assay; the overall concordance with core/NS5B sequencing results was 86.9% for nonselected gt 1 and gt 6 samples (18). Furthermore, Quer et al. reported a gt 1 subtyping rate of 84% for the INNO-LiPA v2.0 assay, while the remaining samples were reported as gt 1 without subtype (15). These reported results are in a range similar to that observed for the Plus assay in our study. It should be noted, however, that we investigated a preselected challenging collection of specimens that included a considerable number of gt 1 samples with subtypes other than gt 1a or 1b, as well as gt 6 samples, which were difficult to distinguish from gt 1, in conformity with the publication by Mallory et al. (14).

Also sequencing, although being considered the gold standard, has limitations in terms of cumbersome protocols and amplification failures. Recently, Cai et al. observed amplification rates of 92.7% and 56.4% for core region and NS5B sequencing, respectively, varying between 16.7 and 100% across viral loads and target regions (18). In the present study, 87.9% of the samples could be amplified for NS5B sequencing and 92.9% in either the NS5B or 5′ UTR/core region. Viral load results were available for 4 of the 7 samples that failed amplification and ranged between 3.78 and 4.51 log IU/ml. Besides potential polymorphisms, this might be considered a reason for failure.

Since completion of this study, Abbott updated the software algorithm of the GT II assay to allow genotype 1 subtyping even in the case of a delayed subtype amplification curve. This will reduce the number of gt 1 samples without subtype and avoid the need for supplementary testing.

Overall, we showed here that the automated Plus assay, which was developed by Abbott Laboratories for further resolution of unsubtypable gt 1 results and future commercialization, allowed accurate genotype/subtype assignment, with high concordance with reference NS5B or 5′-UTR/core region sequencing results, by identifying 1a, 1b, 6, and non-gt 1a/1b/6 genotypes and subtypes. This resolution rate is in the same range as that for supplemental testing by sequencing. Considering that less than 5% of routine samples present a result of gt 1 without subtype, the GT II test in combination with the Plus assay as a second-tier assay may allow successful genotyping of >99% of routine samples.

The high genetic variability of HCV remains challenging for genotype and subtype assignment by commercial assays but also for sequencing, and no perfect method currently exists. Therefore, for the remaining equivocal samples, use of an additional assay is beneficial. The real-time PCR-based Plus assay investigated here is able to resolve successfully gt 1 results without subtype from the GT II assay through specific identification of subtypes 1a and 1b as well as gt 6 reassignment; therefore, it is an easy-to-implement alternative to sequencing-based approaches for supplementary testing.
